# Corrosion Resistance of MgO and Cr_2_O_3_-Based Refractory Raw Materials to PbO-Rich Cu Slag Determined by Hot-Stage Microscopy and Pellet Corrosion Test

**DOI:** 10.3390/ma15030725

**Published:** 2022-01-18

**Authors:** Maciej Ludwig, Edyta Śnieżek, Ilona Jastrzębska, Ryszard Prorok, Yawei Li, Ning Liao, Mithun Nath, Jozef Vlček, Jacek Szczerba

**Affiliations:** 1 Faculty of Materials Science and Ceramics, AGH University of Science and Technology, Al. Mickiewicza 30, 30-059 Kraków, Poland; e.sniezek@wp.pl (E.Ś.); ijastrz@agh.edu.pl (I.J.); rprorok@agh.edu.pl (R.P.); jszczerb@agh.edu.pl (J.S.); 2 Forglass Sp. z.o.o., Wadowicka 8a, 30-415 Kraków, Poland; 3 The State Key Laboratory of Refractories and Metallurgy, Wuhan University of Science and Technology, Wuhan 430081, China; liyawei@wust.edu.cn (Y.L.); liaoning@wust.edu.cn (N.L.); mithunnath@wust.edu.cn (M.N.); 4 National-Provincial Joint Engineering Research Center of High Temperature Materials and Lining Technology, Wuhan 430081, China; 5 Department of Thermal Engineering, Faculty of Materials Science and Technology VŠB, Technical University of Ostrava, 708 00 Ostrava, Czech Republic; jozef.vlcek@vsb.cz

**Keywords:** corrosion, MgO, Cr_2_O_3_, refractory, raw materials, Cu, slag, XRD, SEM

## Abstract

Chemical resistance of commercial refractory raw materials against Cu slag is critical to consider them as candidates for the production of refractories used in Cu metallurgy. In this study, we show the comparative results for the corrosion resistance of four commercial refractory raw materials—magnesia chromite co-clinkers FMC 45 and FMC 57, PAK, and fused spinel SP AM 70—against aggressive, low-melting PbO-rich Cu slag (Z1) determined by hot-stage microscopy (up to 1450 °C) and pellet test (1100 and 1400 °C). Samples were characterized after the pellet test by XRD, SEM/EDS, and examination of their physicochemical properties to explore the corrosion reactions and then assess comparatively their chemical resistance. Since many works have focused on corrosion resistance of refractory products, the individual refractory raw materials have not been investigated so far. In this work, we show that magnesia chromite co-clinker FMC 45 exhibits the most beneficial properties considering its application in the production of refractories for the Cu industry. Forsterite (Mg_2_SiO_4_) and güggenite (Cu_2_MgO_3_) solid solutions constitute corrosion products in FMC 45, and its mixture with slag shows moderate dimensional stability at high temperatures. On the other hand, the fused spinel SP AM 70 is the least resistant to PbO-rich Cu slag (Z1); it starts to sinter at 970 °C, followed by a fast 8%-shrinkage caused by the formation of güggenite solid solution in significant amounts.

## 1. Introduction

The lifetime of refractory lining is one of the most critical factors during high-temperature processes. The repairs of the lining are both time-consuming and expensive, so high-quality refractories are desired and consistently developed. Refractory materials used in the Cu industry are not exposed to extremely high temperatures. However, they have to resist major thermal shocks. Moreover, they are subjected to chemical interactions with the surrounding environment, mechanical wear due to the movement of the stove charge, induced mechanical stresses in the linings, and hot erosion [1]. Refractory linings should withstand the influence of Cu, Cu matte, Cu slag, and SO_2_. Melted Cu does not wet the ceramics; thus, it slightly affects the lifetime of the refractory. However, Cu slag is one of the most aggressive corrosive mediums towards refractories [2]. All factors—mechanical, thermal, and chemical stresses—exist simultaneously during pyrometallurgical Cu production [3,4,5], which intensifies the degradation of the refractory. Cu slag, composed of numerous oxides (Table 1), is generated during the smelting of Cu matte in shaft kilns and converters.

During high-temperature process, the oxide components of the slag form low melting phases as a result of chemical reactions, e.g., fayalite Fe_2_[SiO_4_] (T_M_ = 1205 °C) [8,9], cuprite Cu_2_O (T_M_ = 1215 °C) [10], magnetite Fe_3_O_4_ (T_M_ = 1539 °C) [11], and lead silicate Pb_2_SiO_4_ (T_M_ = 747 °C) [7]. Therefore, since the 1950s, the most suitable refractories for Cu production have been magnesia–chromite refractories produced from chromite ore and sintered or fused magnesia. The magnesia–chromite refractories are fabricated at high temperatures from 1550 °C (silica-bonded) up to 2500 °C (directly bonded; produced by fusion of chromite grains and magnesia clinker) [1,5,12]. The former type of refractory shows excellent resistance in contact with Cu slag [13]; however, it contains Cr^3+^, which tends to oxidize to carcinogenic Cr^6+^. This phenomenon is accelerated when Cr-containing refractories are exposed to temperatures above 800 °C and contact with alkali or alkali earth oxides, especially CaO. Due to this fact, magnesia–chromite was completely withdrawn from the cement, lime, and glass industry [12]. Recycling of spent magnesia–chromite products containing hazardous Cr^6+^ is technologically complex, expensive, and economically not viable [1,14]. Moreover, their landfilling makes it challenging to properly protect and ensure both human and natural environment safety.

In recent years, many researchers have focused on the examination and enhancement of magnesia–chromite (MgO-Cr) refractories [15,16,17]. Chen et al. [2] compared the behavior of a direct-bonded MgO-Cr refractory and fused-grains-based refractories in contact with Cu-Cu_x_O-PbO-based slag. The infiltration of the examined corrosive medium was found to be greater for the fused refractory product. The wetting behavior of chromite grains was found to be the most important factor in the corrosion protection of both types of refractories. The larger fused grains were a fair degree more resistant to corrosion. Other authors revealed the behavior of magnesia–chromite refractories against fayalite slag [18,19,20,21,22,23], Cu matte [21,24], fayalite-based slag with increased ZnO content [25,26,27], or calcium silicate slags [28]. In [23,29], authors presented the reduction of chromium oxide amount in refractories for the Cu industry. Currently, numerous works focus on the development of Cr-free refractories for Cu metallurgy. Jiang et al. [30] investigated the corrosion behavior of MgO-MgAl_2_O_4_-based refractories (M-MA refractories), produced at 1580 °C, against Cu, Cu_2_O, and Cu matte. Cu did not infiltrate into MgO-MgAl_2_O_4_ refractories, and it only gathered on the surface of spinel grains, which confirms the non-wetting behavior of Cu towards the refractory Cu. In addition, MA showed relatively good resistance in contact with Cu matte. A slight reaction rate was observed for the system MgAl_2_O_4_-Cu matte, for which two layers were formed, but, beneficially, Al_2_O_3_ existed in greater amounts in observed layers. The worst corrosion resistance was observed for the interaction between M-MA refractory and Cu_2_O. Cuprite penetrated deeply into the refractory and formed Cu-containing non-stoichiometric compound 2(Mg,Cu_2_)O·3Al_2_O_3_, which was reported for the first time, and easily peeled off from the refractory body. The resistance of MA to liquid CuO_x_ was examined in [31] and to PbO-rich slag in [32]. Petkov et al. [14] compared the chemical behavior of six different Cr-free refractories with six different MgO-Cr refractories. Anode slag was the corrosive medium, which consisted of 50 wt.% CuO_x_, 30–35 wt.% FeO, and 7–8 wt.% SiO_2_. Two of these refractories (MgO-based brick with the addition of ZrSiO_4_ and fused Al_2_O_3_-based brick) revealed extremely high rates of corrosion. Moreover, the addition of spinel (MA) did not improve the corrosion resistance of MgO-based bricks. Consistently, magnesia–chromite refractories exhibited the best performance. Cr-free solutions for the Cu industry are continuously under investigation, e.g., refractories from the system MgO-Al_2_O_3_-SnO_2_ [33,34], Si_3_N_4_-SiC [35], or MgO doped with ZrO_2_ nanoparticles [36]. The resistance of MgO to liquid CuO_x_ was examined in [37].

The influence of PbO-rich non-ferrous slag [38,39,40,41] and PbO-rich ferrous slag [38,41], or PbO-CuO_x_-Cu mixture [2], on the corrosion resistance of MgO-Cr refractories is well recognized in the literature. In our recent work, we concluded that the higher the concentration of PbO in the lead-rich copper slags, the higher the degradation level of magnesia–chromite refractory [42]. However, the chemical interactions between crucial refractory raw materials with slag enriched in PbO and CuO_x_ are poorly investigated so far. Therefore, this work aimed to study the chemical interactions between commercial Cr-containing (magnesia–chromite co-clinkers FMC 45 and FMC57 as well as chromium ore PAK) and Cr-free refractory raw material (fused magnesia–aluminate spinel SP AM 70) against commercial PbO-rich Cu slag (Z1) and identify the most corrosion-resistant one.

## 2. Materials and Methods

The experiment consisted of three main stages: the chemical and structural characterization of four refractory raw materials and Cu slag, preparation of the samples for corrosion tests (refractory raw materials–Cu slag mixtures), and corrosion tests of mixtures by hot-stage microscopy (HSM) and pellet test (PT).

### 2.1. Characterization of Refractory Raw Materials and Cu Slag

Refractory raw materials used in the experiment were the following:

• Magnesia–chromite co-clinkers, FMC 45 and FMC 57

A semi-product for the production of magnesia–chromite co-clinkers is magnesia clinker, which is fabricated by burning magnesia carbonates at 1500–2000 °C and typically contains 95.0–99.8 wt.% MgO. Then, by burning the mixture of MgO clinker and chromite ore in the rotary kiln, or by fusion in an electric arc furnace, the magnesia–chromite co-clinkers are produced [6,43]. In addition to MgO and Cr_2_O_3_, the secondary components in magnesia–chromite co-clinkers are CaO, SiO_2_, and Fe_2_O_3_ in a small percentage of contents. Details are provided in Table 2.

• Pakistani chromite ore, PAK

The phase composition of chromite ores is represented by a complex spinel solid solution (FeO,MgO)·(Cr_2_O_3_,Al_2_O_3_,Fe_2_O_3_), containing 32–50 wt.% Cr_2_O_3_ [43].

Listed raw materials are commonly used during the fabrication of magnesia–chromite refractories applied as linings in slag zones of heating devices for the production of copper.

• Fused spinel, SP AM 70

Magnesia–aluminate spinel is a synthetic raw material produced by arc melting of magnesia and alumina. MgAl_2_O_4_ spinel is characterized by high refractoriness (T_m_ = 2122 °C [44]), a relatively high density of 3.58 g/cm^3^, and a low thermal expansion coefficient of 8.83 × 10^−6^ 1/K (in the range 20–1200 °C) [45]. This material is applied in cement [46,47,48] and the steel industry [49,50,51,52].

The corrosive agent used in the experiment was industrial PbO-rich Cu slag, obtained after the first stage of the converting process. The examined raw materials and slag were characterized in terms of their chemical and phase composition on samples with grain size <63 µm, applying X-ray diffraction (XRD) and X-ray fluorescence (XRF) techniques. The diffraction patterns were collected by applying Philips PANalytical X’Pert-Pro diffractometer at room temperature, in the 2θ range of 5–90°, using a goniometer of 240 mm in diameter and Cu-Kα radiation. Chemical composition was measured using a PANalytical WDXRF Axios mAX spectrometer.

Samples of raw materials and slag used for chemical analysis (by XRF) and phase analysis (by XRD) as well as for both corrosion tests applied in the present study were powdered to below 63 μm. In the first step, bigger pieces of raw materials and slag (above 3 cm) were crushed using a laboratory jaw crusher. Then, they were subjected to a milling process using a centrifugal ball mill with ceramic grinders for 4 h. After this step, each component was sieved using a sieve with a mesh size of 63 μm, and oversized grains were manually milled using agate mortar. Eventually, the powders were mixed for 1 h.

The high-temperature behavior of the slag was tested by hot-stage microscopy (HSM) test to determine its sintering, melting, and flow temperatures and in situ dimensional changes, from room temperature to 1000 °C with a heating rate of 10 °C/min applying Carl Zeiss MH02 microscope. Sintering temperature was determined as corresponding to 2% shrinkage of linear sample dimensions. HSM method was described in detail in works [53,54].

### 2.2. Corrosion Tests of Raw Material–Slag Mixtures

#### 2.2.1. Hot-Stage Microscopy

The powdered mixtures of raw materials and slag in mass ratio 1:1 were homogenized for 1 h and shaped into 3 mm cuboidal samples. Measurements were conducted using Carl Zeiss MH02 microscope with a heating rate of 10 °C/min to 1500 °C. Characteristic temperatures—sintering, melting, and flow—were determined based on in situ changes of the sample cross-section in microscopic view recorded by the camera during heating.

#### 2.2.2. Pellet Test

The pellet test was conducted to identify and characterize the quasi-equilibrium reactions between the powdered mixture of refractory raw materials and Cu slag. Each refractory raw material was separately mixed and homogenized with Cu slag, ground below 63 µm, in the ball mill for 2 h in the mass ratio of 3:1 raw material to slag. The mixtures were uniaxially pressed under 65 MPa in cylindrical steel molds of 20 mm in diameter. The shaped samples were heat-treated in an air atmosphere at 1100 and 1400 °C in the laboratory electric furnace with a heating rate of 5 °C, soaked 3 h at maximum temperature, and finally cooled freely with the furnace. The temperature of 1100 °C was selected to evaluate the changes in phase composition of the samples before reaching a working temperature of copper converter (1300 °C), which can affect the performance of samples’ components.

After the pellet test, the samples were subjected to examination of their bulk density and open porosity using a vacuum impregnator, based on the Archimedes’ principle method. The XRD measurements for identification of the phase composition changes in corroded samples were conducted using Philips PANalytical X’Pert-Pro diffractometer at room temperature with 240 mm goniometer diameter applying Cu-Kα radiation, in the 2θ range of 5–90°. The microstructural analysis of the corroded samples was performed by a scanning electron microscope Nova Nano SEM 200 equipped with an energy-dispersive spectrometer (EDS) to measure the chemical composition in microareas. The samples for microscopic observations were prepared by embedding the material in the epoxy resin, polishing by standard ceramic technique, and coating the surface with a carbon layer.

## 3. Results

### 3.1. Characterization of Raw Materials

#### 3.1.1. Chemical Composition of Raw Materials by XRF

According to Table 2, magnesia–chromite co-clinkers—FMC 45 and FMC 57—consisted mainly of MgO, Cr_2_O_3_, Fe_2_O_3_, and Al_2_O_3_. In FMC 45, MgO reached about 52%, while Cr_2_O_3_ content was under half that amount at 25%; other oxides were present in lower amounts, such as Fe_2_O_3_ at 15% and Al_2_O_3_ at 6%, while the range of the other oxides was below 1%. FMC 57 contained a higher quantity of MgO, equaling 62%. Simultaneously, FMC 57 was characterized by a lower content of Cr_2_O_3_, Fe_2_O_3_, and Al_2_O_3_ at 19%, 10%, and 5%, respectively. PAK contained about 50% Cr_2_O_3_, while the content of oxides MgO, Fe_2_O_3_ Al_2_O_3_, and SiO_2_ reached 17%, 16%, 12%, and 4%, respectively. Fused spinel, SP AM 70, was characterized by contents of MgO and Al_2_O_3_ close to their amounts in the composition of stoichiometric MgAl_2_O_4_ [43]. The impurities in this Cr-free raw material existed mainly as CaO, SiO_2_, and V_2_O_5_, equaling 0.9% in total.

The low content of SiO_2_ and CaO in both magnesia–chromite co-clinkers <1.5% indicates their high suitability for refractory applications. On the other hand, in PAK the total content of Cr_2_O_3_ and Al_2_O_3_ was over 60% [55], and SiO_2_ was slightly higher than recommended for high-temperature applications (<3.5 wt.%) [13]. In addition, SP AM 70 was characterized by a low amount of impurities. Thus, all test commercial raw materials were initially proposed as suitable for application in the Cu industry [43].

#### 3.1.2. The Phase Composition of Raw Materials by XRD

**Figure 1:**
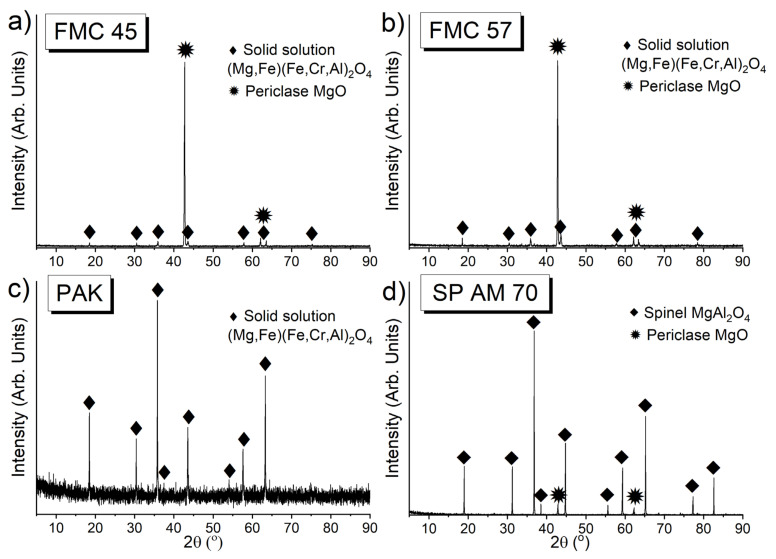
XRD patterns of (**a**) FMC 45, (**b**) FMC 57, (**c**) PAK, (**d**) SP AM 70.

XRD patterns of the test raw materials are presented in Figure 1. The main phase in both magnesia–chromite co-clinkers FMC 45 and FMC 57 as well as in chromite ore PAK (the only phase) was complex spinel solid solution, (Mg,Fe)[Fe,Cr,Al]_2_O_4_, where () and [] indicate tetrahedral and octahedral sites, respectively. MgO was the second phase in FMC 45, FMC 57, and fused spinel SP AM 70. No impurities were detected in all tested samples. In the PAK sample, the increased background is ascribed to the raised iron content among all tested samples (Table 2). This phenomenon was also observed elsewhere [56,57].

### 3.2. Characterization of Cu Slag

#### 3.2.1. Chemical Composition of Cu Slag by XRF

XRF chemical composition of Cu slag is shown in Table 3. PbO was the main component of the test slag, reaching about 40%. Due to the low melting point of PbO (897 °C) [7], it is highly corrosive towards refractory components and, additionally, reduces the viscosity of liquid slag [2]. Other oxides in the test slag were Fe_2_O_3_, CuO, and SiO_2_, which existed in relatively high and comparable amounts of 18%, 16%, and 14%, respectively. The slag was contaminated by CaO and As_2_O_3_ in comparable amounts (3%), as well as by Al_2_O_3_ and ZnO (1.5%). In addition, low amounts of other oxides were detected such as Co_3_O_4_ (1.18%), Na_2_O (0.97%), K_2_O (0.29%), NiO (0.31%), SnO_2_ (0.12%), and Cr_2_O_3_ (0.27%). Typically, Cu slags contain high levels of SiO_2_ and Fe_x_O_y_ [58,59]; hence, the properties of the selected slag in this work were predicted to be distinct. Compared to already investigated PbO-rich slags [38,41], the present slag is distinguished by a high level of aggressive Cu oxide.

#### 3.2.2. The Phase Composition of Cu Slag by XRD

**Figure 2:**
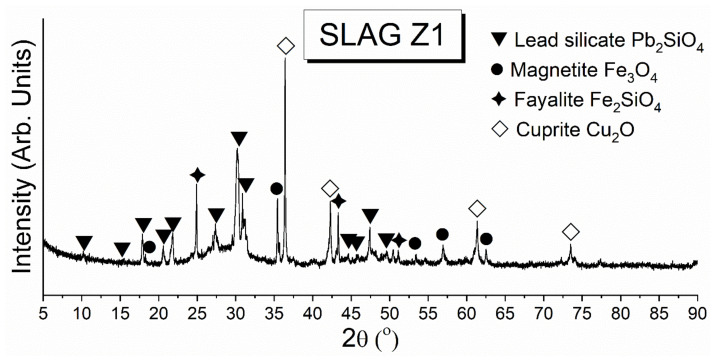
XRD pattern of the test slag [42].

Figure 2 depicts the phase composition of the test PbO-rich copper slag. Pb_2_SiO_4_ was the main phase of this slag, resulting from the high content of PbO and SiO_2_. Other phases present in the slag were magnetite, Fe_3_O_4_, fayalite, Fe_2_SiO_4_, and cuprite, Cu_2_O. Moreover, the increased background at the XRD pattern indicates that the slag contained a glassy phase, resulting from the fast cooling of the test slag [59].

#### 3.2.3. Hot-Stage Microscopy Test of Cu Slag

**Figure 3:**
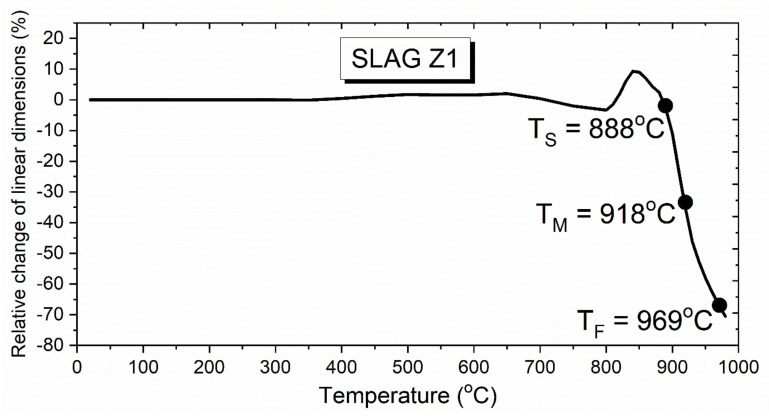
Results of hot-stage microscopy test of test slag; T_S_-sintering temperature, T_M_-melting temperature, T_F_-flow temperature [42].

Figure 3 presents the relative change in the linear dimension of the Cu slag cuboid sample as heated during the hot-stage microscopy test. During heating up in the range of 350–650 °C, the sample slightly expanded, as seen by the rising curve. Then, after the sharp descending stage, a second expansion in the narrow temperature range of 800–850 °C was observed. Finally, the sample started to sinter at 888 °C, followed by melting at 918 °C and full flow at 969 °C. The slag melted at a significantly lower temperature compared to typical fayalite-based slags (1420 °C in air [60] and above 1420 °C [61]) due to high Pb and Cu content.

### 3.3. Corrosion of Raw Material–Cu Slag Mixtures

#### 3.3.1. Hot-Stage Microscopy Test

**Figure 4:**
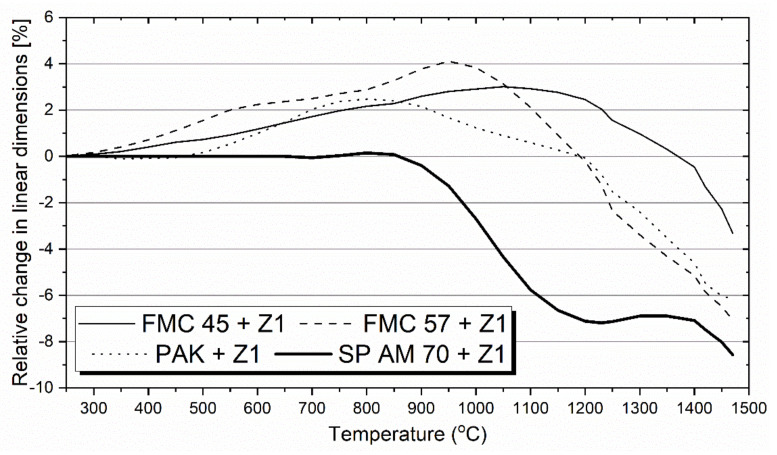
Results of hot-stage microscopy test for refractory raw material–Cu slag mixture. The sintering temperature was set as 2% linear shrinkage of the test sample.

Figure 4 depicts the hot-stage microscopy test results of the refractory raw materials FMC 45, FMC 57, PAK, and SP AM 70 mixed with PbO-rich Cu slag. The pictures of in situ changes in samples cross-sections are presented in Table 4. The highest sintering temperatures were observed for the raw materials containing high levels of Cr_2_O_3_—both magnesia–chromite co-clinkers and PAK, with the highest sintering point registered for FMC 45 of 1440 °C containing 25% Cr_2_O_3_. Such a high sintering temperature can be an effect of optimal content ratio Cr_2_O_3_/MgO (Table 2). Pakistani chromite ore PAK and magnesia–chromite co-clinker FMC 57 showed comparable sintering points of 1285 °C and 1260 °C, respectively. PAK is characterized by the highest Cr_2_O_3_ content of about 50% and, simultaneously, by the lowest MgO amount of 17% among all tested raw materials.

The presented curves (Figure 4) show the expansion for all test raw materials. The most significant linear expansion of 4% was observed for the mixture of FMC 57 + slag at about 950 °C. This resulted from the highest level of MgO, which possesses a high thermal expansion coefficient (α_MgO_ = 15.60 × 10^−6^ 1/K [62]). FMC 45 + slag reached a maximum expansion of about 3% at 1100 °C; for PAK, the maximum expansion was registered at 800 °C as 2.5%.

Conversely, no-chrome raw material—fused spinel SP AM 70—showed steady dimensions up to 850 °C, followed by very sharp shrinkage from 850 °C up to 1250 °C, reaching 7%. The dimensions were maintained up to 1400 °C, above which the sample started to shrink again. Additionally, SP AM 70 revealed the lowest sintering point of 970 °C and, through this test, was confirmed to be the least resistant to PbO-rich Cu slag. For this material, only shrinkage was observed. In contrast to the fused spinel (SP AM 70), PAK expanded much more during heating, which is typical for Cr-containing spinels. Notably, FeCr_2_O_4_ is characterized by a high thermal expansion coefficient of 12.38 × 10^−6^ 1/K (close to the thermal expansion coefficient of MgO) when compared to the thermal expansion coefficient of spinel *sensu stricto* of 8.83 × 10^−6^ 1/K (all coefficients were given for temperature range 20–1200 °C) [62]. None of the test mixtures started to flow when reaching the limit temperature of the test of 1500 °C.

#### 3.3.2. Pellet Test

##### Phase Composition of the Raw Material–Slag Mixtures after the Pellet Test

**Figure 5:**
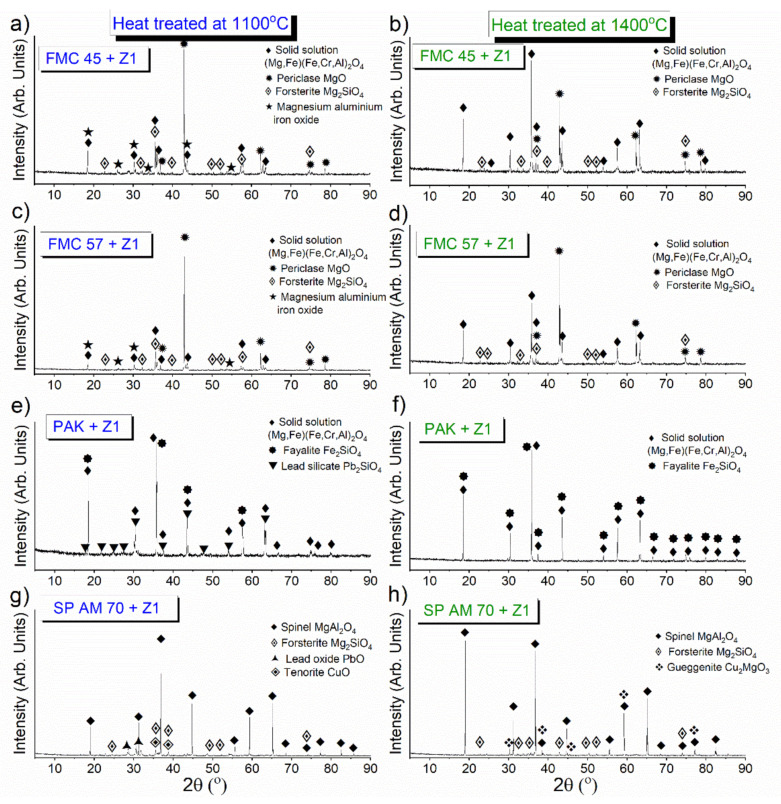
XRD patterns of raw material–slag mixtures after heating at 1100 °C—(**a**) FMC 45 + Z1, (**c**) FMC 57 + Z1, (**e**) PAK + Z1, (**g**) SP AM 70 + Z1 and 1400 °C—(**b**) FMC 45 + Z1, (**d**) FMC 57 + Z1, (**f**) PAK + Z1, (**h**) SP AM 70 + Z1.

Figure 5 shows that both magnesia–chromite co-clinkers—FMC 45 and FMC 57—showed the same phase composition after corrosion by pellet test at 1100 and 1400 °C. The new phases, which appeared at a lower temperature of 1100 °C, were forsterite, Mg_2_SiO_4_, and spinel solid solution, identified as a separate spinel phase probably due to different stoichiometry compared to the original spinel, as its reflexes are located just next to the original spinel ones. This phase disappeared at 1400 °C.

In PAK, for corrosion at 1100 °C, two new phases formed, fayalite (Fe_2_SiO_4_) and lead silicate (Pb_2_SiO_4_), while the former disappeared when heating the mixture at 1400 °C. The clear phase identification of solution phases by XRD is very limited, as the method does not identify the compounds directly but only their crystalline structure by the position of the peaks. The replacement of one element by another in a solid solution may shift the lattice parameters and consequently the position of the peaks.

Corrosion of fused spinel SP AM–slag mixture at 1100 °C showed a more significant number of corrosion products: forsterite (Mg_2_SiO_4_), PbO, and CuO. Copper (II) oxide—tenorite—was detected (instead of Cu_2_O) due to oxidizing atmosphere, as the investigation was done after cooling. According to the CuO-Cu_2_O-MgO phase diagram [63], below 1021 °C, the phase transformation Cu_2_O→CuO occurs. However, heating at a higher temperature of 1400 °C resulted in the CuO transformation into güggenite (Cu_2_MgO_3_) due to a direct reaction of tenorite with magnesia. Moreover, PbO disappeared during heating at 1400 °C as a result of its dissolution in other phases at increased temperatures.

In summary, all of the samples underwent active corrosion without the formation of any corrosion-protecting interfaces. At 1400 °C, forsterite (Mg_2_SiO_4_) was identified in FMC 45, FMC 57, and SP AM 70 (which possess above 20% of MgO), while fayalite (Fe_2_SiO_4_) was detected in PAK, which contains a significant iron content (16% of Fe_2_O_3_). Nevertheless, those two compounds create a continuous solid solution, called olivine, as per the diagram Mg_2_SiO_4_-Fe_2_SiO_4_ [64]. Endmembers detected by XRD are in fact solid solutions where Mg and Fa can replace each other more or less arbitrarily or be substituted by other elements of similar geometry such as Ca, Zn, or Ni. There are works [65] showing that even large-size Pb can substitute for Mg in oxide structure despite the great difference in cation radii (r_Pb2+_ = 119 pm, r_Mg2+_ = 72 pm); however, very scarce information on the system MgO-PbO exists in literature (and a low number of cards are available in crystallographic databases). In the discussed system Mg_2_SiO_4_-Fe_2_SiO_4_, the increased amount of Fe leads to a continuously descending melting point of the solid solution from 1890 °C for forsterite to 1205 °C for fayalite. Table 5 shows summarized phases detected using XRD method.

##### Bulk Density and Open Porosity of the Raw Material–Slag Mixtures after Pellet Test

**Figure 6:**
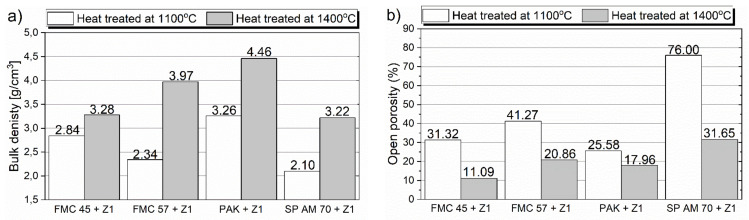
Bulk density (**a**) and open porosity (**b**) of samples after pellet corrosion test at 1100 °C and 1400 °C.

Results of bulk density and open porosity for corrosive raw material–slag mixtures after pellet test are presented in Figure 6. The greatest density was detected for chromite ore PAK, being 3.3 g/cm^3^ and 4.5 g/cm^3^ for 1100 and 1400 °C, respectively. This behavior agreed with the low porosity of 25.6% for 1100 °C and 18.0% for 1400 °C. Out of the mixtures with magnesia raw materials, the purer FMC 57 showed a lower density at 1100 °C of 2.3 g/cm^3^ than FMC 45. However, after heating at 1400 °C, it increased about 70% to 4.0 g/cm^3^, which significantly exceeded the density of FMC 45 of 3.4 g/cm^3^. Nevertheless, FMC 45 + slag reached the lowest porosity of 11.1% at 1400 °C, while for FMC 57 + slag, it equaled 20.9%.

The slag mixture with spinel SP AM 70 exhibited the lowest density at 1100 and 1400 °C of 2.1 g/cm^3^ and 3.2 g/cm^3^, respectively. This behavior was accompanied by the extremely high porosity of 76% at lower temperatures and 31.7% at higher test temperatures. Such porosity can be associated with a high MgO diffusion rate from the spinel into slag at 1100 °C, leaving pores, which preceded güggenite formation, confirmed by XRD at 1400 °C (Figure 5h).

The results show the positive influence of Cr_2_O_3_ content on the high densification of the material linked with higher compactness of the sample represented by low open porosity. Moreover, the impurities in magnesia also lead to greater densification of the sample. This trend may influence lower penetration of materials by liquid slags present at operation conditions.

##### ***Microstructure Analysis of the Raw*** ***Material–Slag Mixtures after the Pellet Test***

**Figure 7:**
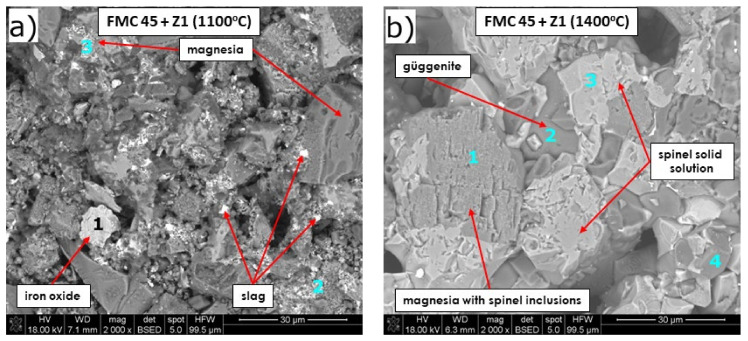
SEM images of magnesia–chromite co-clinker FMC 45 after pellet test at (**a**) 1100 °C, (**b**) 1400 °C.

SEM microphotographs of FMC 45 corroded by Cu slag at 1100 and 1400 °C, together with EDS chemical analysis, are presented in Figure 7 and Table 6. Chemical analysis verified XRD results revealing magnesia and complex spinel solid solution in the samples after corrosion (Figure 7b, p. 1 and 3, respectively). For samples treated at 1400 °C, güggenite solid solution formed between magnesia and spinel grains (Figure 7b, p. 2), as a result of a reaction between magnesia from refractory and copper oxide from the slag. MgO grains at 1400 °C (Figure 7b) contained other dissolved elements such as Si (0.2%), Pb (0.8%), Cu (6.1%), and Fe (8.4%). Points 2 and 3 in Figure 7 represent average chemical compositions of magnesia grains containing PbO inclusions at 1100 °C, represented by dispersed small lightest-color microareas due to high molar weight. Scarce information on the system MgO-PbO exists in literature; thus, PbO solubility in MgO needs further study. Chen et al. [66] examined the MgO-PbO-SiO_2_ system in the temperature range 700–1400 °C and found no phases containing only PbO and MgO, but they found three stable phases in the system PbO-SiO_2_ (PbSiO_3_, Pb_2_SiO_4_, and Pb_4_SiO_6_) and one ternary compound (Pb_8_Mg(Si_2_O_7_)_3_). Najem et al. [65] recently doped MgO nanoparticles with Pb^2+^, but the maximum lead amount doped was low, from 0.03 to 0.05 at.%. X-ray diffractometry confirmed that only the cubic phase of space group Fm3m was generated, characteristic of MgO. The increased Pb^2+^ fraction doped in MgO caused the most intensive reflex (200) shift towards the lower 2θ values, and the increase in lattice parameter and crystallinity was observed. Moreover, the intensity of the most substantial peak decreased, attributed to the difference in the ionic radius between Pb^2+^ (r_Pb2+_ 119 pm) and Mg^2+^ (r_Mg2+_ = 72 pm).

Spinel solid solutions were found to accept various stoichiometries and numerous ions into their structure (XRD showed secondary spinel as a separate phase). At 1400 °C, grains of spinel solid solution, as shown by point 3 in Figure 7b, were enriched in Mg (16%) and Fe (21%) with a relatively low content of Cr (11%), compared to other locations presented by point 4 where they were enriched with Cr (17%) and impoverished concerning Fe (16%). In addition, it can be seen that spinel solid solution contained low substitutions of Pb at the level of 1%. Similar to the binary system MgO-PbO, the solubility of Pb^2+^ in spinel solid solution in chromite ore was not found in the literature. Forsterite was not observed in SEM images, although it was identified by XRD, probably due to its low quantity in the sample.

Arsenic ions were detected only at 1400 °C in MgO (2%: point 1, Figure 7b) and in spinel solid solution grain (3%: point 4, Figure 7b).

The sample FMC 45 after corrosion at 1400 °C was visibly more densified compared to 1100 °C, with almost no slag phase in between the grains, which confirms the results showing its greater density and lower open porosity (Figure 6).

**Figure 8:**
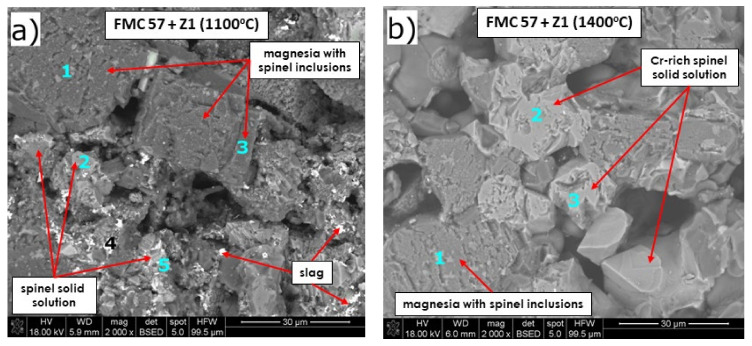
SEM images of magnesia–chromite co-clinker FMC 57 after pellet test at (**a**) 1100 °C and (**b**) 1400 °C.

In FMC 57 for both 1100 and 1400 °C (Figure 8 and Table 7), magnesia and complex spinel solid solution were detected, which confirms XRD results of the corroded mixtures (Figure 5c,d). Pb from slag diffused and dissolved into the structure of complex spinel solid solution in low amounts at 1100 °C of 1% in p. 2 (Figure 8a) and 0.4% in p. 2 (Figure 8b) at 1400 °C.

Among main slag components, MgO accepted Cu more than Pb—greater amounts of Cu dissolved at 1100 °C (5.5%: p. 1; 3.4%: p. 3; Figure 8a) than at 1400 °C (0.7%, p. 4, Figure 8a). Slightly higher amounts of Cu dissolved in spinel solid solution (2.7%—1100 °C, 0.7%—1400 °C).

Spinel solid solution grains detected in the material contained different amounts of constituent ions, e.g., at 1400 °C spinel grains mainly differed in the content of Cr and Fe (points 2,3 in Figure 8b). After treatment at 1400 °C, the slag phase was no longer visible in SEM images, compared to 1100 °C, at which point slag was distributed at grain boundaries.

Arsenic ions were detected—similarly to FMC 45 + slag—in MgO grains (about 2% in p. 1 and about 1% in p. 4, Figure 8a) and in spinel grains (2% in p. 2, Figure 8b).

**Figure 9:**
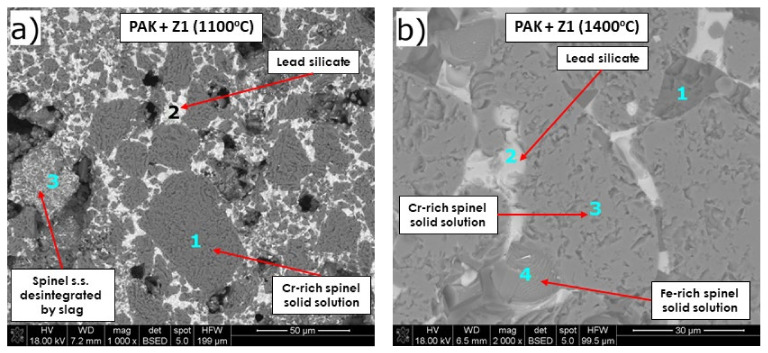
SEM images of PAK after pellet test at 1100 °C (**a**) and 1400 °C (**b**).

The main phase detected in PAK by SEM/EDS (Figure 9 and Table 8) was Cr-rich complex spinel solid solution, the only phase component in the original PAK (XRD-Figure 1c). This phase was found to accept about 5% of Cu for every measured microarea (points 1,3 in Figure 9a; points 3,4 in Figure 9b). Large grains of chromite spinel solid solution of above 50 µm showed high corrosion resistance against Pb, as lead was not detected in them at 1100 °C (point 1, Figure 9a) and only in slight amounts of 1.7% at 1400 °C (point 4, Figure 9b).

The new phases—corrosion products in the mixture PAK + slag—confirm XRD results: lead silicate Pb_2_SiO_4_ (light grey areas; 1100 °C, 1400 °C: point 2 in Figure 9a,b) and olivine [Mg,Fe]_2_SiO_4_ (dark grey area; 1400 °C: point 1, Figure 9b). The amount of secondary Pb_2_SiO_4_ was significant in SEM images. Pb_2_SiO_4_ dissolved comparable, low amounts of Mg^2+^ and Cr^3+^ of about 2–3%. 2.5% of arsenic ions were registered only in Cr–spinel solid solution (point 1 in Figure 9a; point 3,4 in Figure 9b).

**Figure 10:**
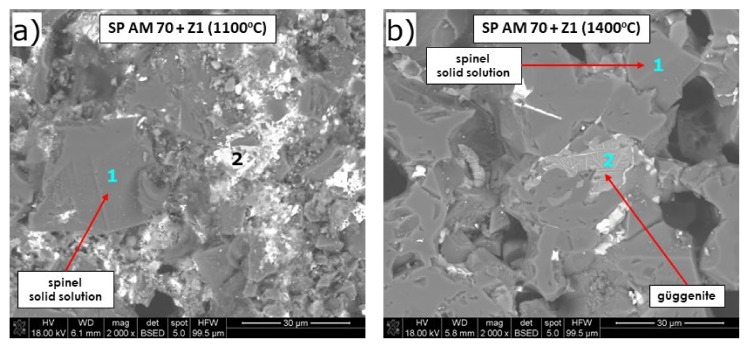
SEM images of fused spinel SP AM 70 after pellet test at 1100 °C (**a**) and 1400 °C (**b**).

SEM/EDS analysis of the test mixture of fused spinel SP AM 70 + slag (Figure 10, Table 9) revealed güggenite as corrosion product at 1400 °C (also identified by XRD—Figure 5h). Güggenite is a non-stoichiometric phase that is stable over a wide range of compositions, as shown by phase equilibria of the MgO-Cu_2_O-CuO system in [63]. Güggenite did not accept any Pb. It dissolved very slight amounts of Cr of 0.2% and Al of 0.4% and an increased amount of Fe of 4.2%. This phase appeared in SEM images as light-grey microareas. It shall be mentioned that there is scarce information in the literature about the properties of this phase. The slag phase, visible as light-grey microareas (p. 2, Figure 10a), was enriched with Mg (23%), which comes from the dissolution of spinel from refractory by aggressive slag. Si was not detected to greatly infiltrate the spinel phase, as maximum Si content in spinel grains was 0.3% at 1100 °C, and it increased to 5.1% at 1400 °C.

Spinel phase MgAl_2_O_4_ showed relatively high resistance to Pb-rich copper slag at high temperatures, as only about 0.7% of Pb and 0.6% of Cu were detected in the spinel grains at 1100 °C, while at 1400 °C only Cu was observed at the level of 1.3%, and no Pb was detected. Spinel accepted above 5% of Fe at 1400 °C (and 0.7% at 1100 °C), and this behavior is characteristic for spinel structure, which tolerates ions with radii 40–80 pm (ferrous ions: r_Fe2+,IV_ = 61.5 pm, r_Fe2+,VI_ = 74 pm; ferric ions: r_Fe3+,IV_ = 49 pm, r_Fe3+,VI_ = 64.5 pm) [67,68,69,70,71]. Arsenic ions were not registered in any analyzed region.

## 4. Discussion

The study presents comparative results of chemical resistance of four commercial raw materials—two kinds of magnesia–chromite co-clinkers (FMC 45 and FMC 57), chromite ore PAK, and fused spinel SP AM 70—commonly used for the production of refractories. So far outside this study, only refractory products have been investigated in terms of their chemical resistance to copper slags. Table 10 summarizes and compares the results obtained in this work with results obtained for refractory products in other works.

The slag used in the experiment was PbO-rich slag containing about 40% PbO, 18% Fe_2_O_3_, 16% CuO, and 14% SiO_2_, derived from the Cu converting process. The oxides existed in the slag in the form of four main phases—Pb_2_SiO_4_, Fe_3_O_4_, Fe_2_SiO_4_, and Cu_2_O—which are aggressive towards refractory material as they possess low melting points: Pb_2_SiO_4_ at 747 °C [7] Fe_2_SiO_4_ at 1220 °C [72], and Cu_2_O at 1230 °C [73].

The presence of these low-melting-point components affected the high-temperature behavior of the raw materials tested by hot-stage microscopy (HSM), especially fused spinel SP AM 70, which started to sinter at 970 °C followed by drastic shrinkage reaching 7% at 1400 °C. This test showed the low resistance of this kind of raw material to PbO-rich Cu slag and confirmed that it should not be used as the main refractory component for non-ferrous metallurgy where similar slags occur.

The opposite behavior was found for Pakistani chromite ore PAK, which showed high resistance to PbO-rich slag and the best dimensional stability up to 1200 °C (the most crucial temperature range for the copper industry). A similar high-temperature behavior during the HSM test was observed for FMC 45 and 57, although they expanded more. The highest sintering point of 1440 °C, determined as 2% linear shrinkage of the test sample, was registered for the mixture FMC 45 + slag.

Comparing both HSM and pellet corrosion tests, FMC 45 is the most corrosion-resistant against PbO-rich slag among all test raw materials. The new phases that appeared after corrosion in FMC 45 were forsterite (Mg_2_SiO_4_) due to reaction between MgO from refractory and SiO_2_ from slag (acc. to Reaction (1)) and spinel solid solution having different stoichiometry than the original, which disappeared at 1400 °C. Here, FMC 45 revealed extremely low open porosity (11%). Forsterite formed in relatively low amounts in FMC 45 and FMC 57, as it was not observed during SEM/EDS analysis (Figure 7a,b and Figure 8a,b), and this was confirmed by low intensity of reflexes in XRD patterns (Figure 5a,b).

MgO_ref_ + SiO_2slag_ → Mg_2_SiO_4solid solution_ (1)

Although forsterite is a high-temperature phase, melting congruently at 1890 °C [6], thus improving thermal resistance of the material, and possessing good chemical stability and excellent insulation properties, it is simultaneously characterized by a relatively low density (3.21 g/cm^3^). This is lower compared to the rest of the predominant phases—ρ_MgO_ = 3.59 g/cm^3^, ρ_(Fe,Mg)Cr2O4_ = 4.70 g/cm^3^—resulting in the major loosening of the microstructure and causing, if it occurs in excess, so-called “forsterite bursting”, detrimental for the performance of refractories. The microstructure disintegration was previously reported for massive formation of forsterite as corrosion product [2,14,22,39,40,67,74,75].

Aggressive components of the slag such as Cu and Pb dissolved in the MgO grains of FMC 45 and FMC 57. For instance, 2% of Cu dissolved in MgO grains of FMC 45 at 1100 °C, increasing to 6.1% at 1400 °C. For FMC 57, 5.5% of Cu was detected in MgO at 1100 °C. As per the MgO-CuO_x_ phase diagram [63], MgO can dissolve up to 21% of Cu ions at 700 °C (2), maintained until 1048 °C, then decreases to 10% and 5% at 1200 °C and 1400 °C, respectively, which proves the observed results.

MgO_refractory_ + CuO_x,slag_ → (Cu,Mg)O_solid solution_ (2)

Together with Cu, Fe diffused into periclase grains in significant amounts of about 8% (point 1, Figure 7b). This phenomenon is commonly observed [2,3,4,14,25] due to the similar geometry of ions in both oxides (r_Mg2+_ = 72 pm, r_Fe2+_ = 77 pm) [76]. In general, Pb diffused more willingly into Cr–spinel solid solution grains than MgO grains, which was previously observed in our recent work [42] for interactions between MgO-Cr refractory and PbO-rich slags and in [2] for interactions between MgO-Cr refractory and Cu-Cu_x_O-PbO melt. The study on the MgO-PbO system, as well as solubility of Pb^2+^ in Cr–spinel solid solution, requires future study due to a gap in the literature, despite its significance in the industry. Si tends to diffuse mainly to MgO grains, with its greatest level of 5.7% at 1100 °C, but it remained unreacted (dissolved in magnesia). In contrast, Mg diffused into slag, which was observed in FMC57 (26% Mg, p. 4, Figure 8a) or SP AM 70 (24% Mg, p. 2, Figure 10a).

FMC 57 and PAK, although possessing various phase compositions, showed similar high-temperature behavior in the HSM test, with sintering points for both raw materials above 1250 °C, followed by a 6% shrinkage up to the limit temperature of measurement of 1500 °C. Large grains of Cr–spinel solid solution in PAK, especially those enriched with Cr and Fe, showed excellent resistance to Pb infiltration. In contrast, the small ones were surrounded by the corrosion product—low melting phase of non-stoichiometric (as confirmed by SEM/EDS) lead silicate, Pb_2_SiO_4_ (T_m_ = 747 °C [7]), appeared as light-grey microareas. Pb_2_SiO_4_ constituted the main phase component of the original slag identified by XRD; it was generated in slag as a result of a reaction between PbO (slag component) and SiO_2_ (flux) during slag formation in Cu converting (3). This phase existed in equilibrium with the main component of PAK—complex Cr–spinel solid solution—in the conditions of the pellet corrosion test both at 1100 °C and 1400 °C.

2PbO_slag_ + SiO_2,flux or concentrate_ → Pb_2_SiO_4slag_ (3)

Cr–spinel solid solution accepted more Cu than Pb, as the average amount of Cu in spinel grains was 5%, while the content of Pb was about 1.7%. Overall, the amount of Cu accepted by the phases of PAK was doubled when compared to FMC 45. The second component of the slag—fayalite Fe_2_SiO_4_—accepted Mg from PAK and formed olivine solid solution [Mg_,_Fe]_2_SiO_4_, which is commonly found as a corrosion product in MgO-containing refractories when fayalite-based Cu slags accompany the process [14,74]. With the increased content of MgO in the slag or refractory, part of Fe^2+^ (r_Fe2+_ = 74 pm) in Fe_2_SiO_4_ is replaced by Mg^2+^ of smaller ionic radius (r_Mg2+_ = 66 pm), which leads to a decrease in lattice parameter reflected by the overall right shift of XRD reflexes [77]. As long as the MgO is more than FeO in olivine, the melting temperature of solid solution is high [64], and the material is safe for refractory application up to 1350 °C, typical for Cu production. PAK showed relatively high density after corrosion at 1400 °C of 4.70 g/cm^3^, which is the effect of high Cr_2_O_3_ content (ρ_Cr2O3_ = 5.22 g/cm^3^). In contrast to the shrinkage of SP AM 70 during heating, PAK—chromium-containing raw material—expands much during heating, which may result from changing Fe^2+^/Fe^3+^ ratio during heating in the air—a partial reduction of iron oxides in (Mg,Fe)(Fe,Cr,Al)_2_O_4_ up to 1200 °C, followed by their re-oxidation [68].

Fused spinel SP AM 70 showed the lowest corrosion resistance to PbO-rich slag among all tested refractory raw materials. This study showed that the spinel phase of low Cr_2_O_3_ content is poorly resistant to Cu slag. SP AM 70 mixed with slag started to sinter as at 970 °C, which is especially unfavorable due to the fast and significant shrinkage of the material afterward. Such behavior resulted from the massive formation of a new corrosion product—güggenite. Güggenite formed in significant amounts in SP AM 70, as reflected by the high intensity of reflexes in XRD (Figure 5h), and this new phase was also observed by SEM/EDS (Figure 10b). As shown in phase equilibria of the MgO-Cu_2_O-CuO system in [63], güggenite is a non-stoichiometric phase that is stable over a wide range of compositions from CuMgO_2_ to Cu_3_MgO_4_. Güggenite was found to exist in three crystallographic forms, namely, Güggenite A (orthorhombic, Pmmn (59)), güggenite B (orthorhombic, space group I(0)), and güggenite X (monoclinic). Güggenite A (67% CuO, 33% MgO) was obtained [63] in air at 1000 °C, and it was found to transform into güggenite X above 1050 °C. Güggenite B (75% CuO, 25% MgO) was obtained above 1050 °C. Güggenite can exist in equilibria with CuO (tenorite) and Cu_2_O (cuprite); with tenorite (monoclinic) at temperatures below 1000 °C; and with cuprite (cubic) above 1021 °C. This phase was lately shown in MgO-based material corroded by CuO from matte [77]. In our work, the CuO_x_-MgO phase that appeared is characterized by the lower Cu/Mg ratio of 0.7 for FMC45 (p. 2, Figure 7b) and 0.2 for PAK (p. 2, Figure 10b), while theoretically Cu/Mg in güggenite can vary from 1 (for CuMgO_2_) to 3 (for Cu_3_MgO_4_). However, in [63] where authors investigated compositions with the general formula Mg_1-x_Cu_x_O (x = 0 ÷ l.0), güggenite A appeared from x = 0.17 (950 °C), which corresponds to Cu/Mg = 0.2. This confirms the phase observed in the present work of low Cu/Mg ratio is in fact güggenite. Thus, güggenite can occur in a wider composition range, depending on temperature.

As can be seen in the magnified image (Figure 10b), the güggenite phase is likely to separate CuO of needle morphology. The needles-like small crystals were previously found in tenorite by several authors [77]. In fact, the presence of CuO needles in the neighborhood of güggenite can result from the fact that güggenite underwent decomposition during free cooling, as it decomposes at 1062 °C. Similarly, very scarce information is available on the system PbO-MgO, which requires further study.

MgO_refractory_ + 2CuO_slag_ → Cu_2_MgO_3solid solution_ (4)

Cu_2_MgO_3_ was previously found [67] during corrosion of MgO-Cr refractory by slag in the air atmosphere. Güggenite formed during cooling of the sample below 1100 °C; however, its physicochemical properties are poorly recognized and require future studies. According to the ternary system CuO-Cu_2_O-MgO [63], Cu_2_MgO_3_ is stable below 1062 °C. This means that the presence of güggenite is unfavorable in ab refractory, which operates in temperature range 900–1100 °C, as its decomposition above 1062 °C causes spalling of the refractory structure, e.g., during refractory reheating [67]. Moreover, forsterite formed in SP AM 70, but its amount was relatively low, as confirmed by the slight intensity of XRD reflexes characteristic for this phase. Despite corrosion resistance of spinel solid solution grains (Figure 1, Table 9; [31]), discussed phenomena of formation of new phases (forsterite, güggenite) contributed to the overall poor resistance of SP AM 70 to Cu slag. In addition, in [19] authors observed the liquid Cu-slag penetrated throughout the interfaces in a MgO-MgAl_2_O_4_-based refractory.

In the present work, low amounts of As were detected (~2%), which dissolved mostly in MgO and spinel grains. As assumed by Reinharter et al. [78], As_2_O_3_ tend to react mainly with basic components of refractories.

**Figure 11:**
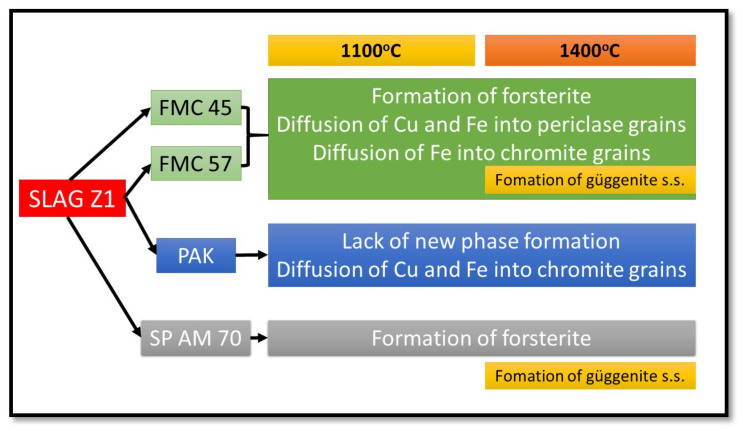
The main mechanisms of the corrosion interaction between refractory raw materials and slag Z1 at 1100 °C and 1400 °C.

Simplified corrosion resistance mechanism was given in Figure 11.

**Table 10:** Comparison of results obtained in the present study with other works. MgO-Cr—magnesia–chromite product, Al_2_O_3_-Cr—alumina–chromite product.

Research Work	Testing Materials and Conditions	Massive Formation of Forsterite	Dissolution of Mg into Melt	Diffusion of Fe and Cu into MgO Grains	Diffusion of Fe into Chromite Grains	Formation of Güggenite in Contact with Cr-Containing Material	Formation of Güggenite in Contact with Cr-Free Material	Formation of Corrosion Protective/Interface Layer
Present work	Raw materials: FMC 45, FMC 57, PAK, SP AM 70 Corrosive agent: PbO-rich copper slag Temperature: 1100 °C and 1400 °C	+	+	+	+	+	+	
[42]	Product: MgO-Cr Corrosive agent: PbO-rich copper slags Temperature: 1300 °C	+	+	+	+	+		
[40]	Product: MgO-Cr Corrosive agent: PbO-based slag Temperature: 1300 °C	+	+		+			+
[39]	Product: MgO-Cr Corrosive agent: PbO-SiO_2_-MgO slag Temperature: 1200 °C	+	+		+			
[2]	Product: MgO-Cr Corrosive agent: Cu-Cu_x_O-PbO Temperature: 1200 °C		+	+	+			
[25]	Product: MgO-Cr Corrosive agent: Fayalite slag with increased ZnO content Temperature: 1200 °C		+		+			+
[74]	Product: MgO-Cr Corrosive agent: Copper smelting slag Temperature: 1250 °C	+	+	+	+	+		
[29]	Product: Al_2_O_3_-Cr Corrosive agent: Fayalite slag with increased ZnO content Temperature: 1200 °C		Dissolution of Al in slag		+			+
[30]	Product: MgAl_2_O_4_ Corrosive agent: Cu_2_OT emperature: 1300 °C							+
[36]	Product: MgO doped with ZrO_2_ nanoparticles Corrosive agent: Fayalite slag Temperature: 1450 °C	+	+					

## 5. Conclusions

- Four commercial refractory raw materials commonly used in refractories for Cu metallurgy—two magnesia–chromite co-clinkers (FMC 45 and FMC 57), a chromite ore (PAK), and a fused spinel (SP AM 70)—were comparatively investigated against PbO-rich Cu slag by hot-stage microscopy and pellet corrosion test.
- Test slag, containing the high levels of PbO of 39% and CuO_x_ of 16%, was characterized by a low melting point of 969 °C, determined by hot-stage microscopy test.
- From the results of both pellet and hot-stage microscopy corrosion tests, the most beneficial behavior was determined for FMC 45. It exhibited relatively stable dimensions during heating with 2% shrinkage, which corresponded to sample sintering at 1440 °C, being the highest sintering point among all tested raw materials. The only corrosion product was forsterite, which formed in slight amounts.
- Fused spinel (SP AM 70) was the least resistant to PbO-rich slag, as it started to sinter as first at 970 °C, followed by a fast and high 8% shrinkage of the material afterward. Güggenite solid solution formed due to the reaction between CuO_x_ from slag and MgO from refractory. This phase is potentially detrimental, as it decomposes above 1062 °C, leading to the spalling of the material during reheating. Moreover, forsterite (Mg_2_SiO_4_) formed during corrosion can be harmful in larger amounts due to its significant volume.
- Despite the different phase composition, PAK and FMC 57 showed comparable corrosion resistance and high-temperature behavior; thus, they constitute the most promising prospective raw materials for refractories dedicated to non-ferrous metallurgy where aggressive Pb-Cu-O slags occur.

## Figures and Tables

**Table 1:** Melting points of oxide components of slags from Cu production [6,7].

Oxide	As_2_O_3_	PbO	CuO	Fe_2_O_3_	SnO_2_	SiO_2_	ZnO	Al_2_O_3_	CaO	MgO
Melting point [°C]	312	897	1085	1565	1630	1723	1975	2020	2625	2825

**Table 2:** Chemical composition (wt.%) of refractory raw materials by XRF.

Oxide	Magnesia–Chromite Co-Clinker FMC 45	Magnesia–Chromite Co-Clinker FMC 57	Pakistani Chromite Ore PAK	Fused Spinel SP AM 70
MgO	51.76	61.89	17.18	27.86
Al_2_O_3_	6.18	5.14	11.69	70.59
Cr_2_O_3_	24.87	19.40	49.76	-
Fe_2_O_3_	15.04	10.48	16.32	0.40
SiO_2_	0.92	1.37	3.91	0.17
CaO	0.76	1.33	0.26	0.69
V_2_O_5_	0.06	0.07	0.09	-
Others	0.41	0.32	0.79	0.29

**Table 3:** Chemical composition of test slag [42].

**Oxide**	PbO	Fe_2_O_3_	CuO	SiO_2_	As_2_O_3_	CaO	Al_2_O_3_	ZnO	Others
**wt. [%]**	39.10	18.20	15.60	14.00	3.35	3.34	1.58	1.18	3.65

**Table 4:** Microscopic view of the test raw material–Cu slag mixtures during the hot-stage microscopy test.

	FMC 45	FMC 57	PAK	SP AM 70
Room temperature	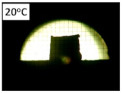	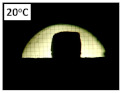	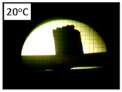	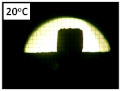
Sintering temperature	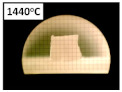	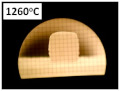	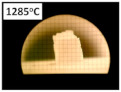	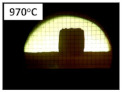
Sample at the end of the test	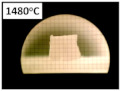	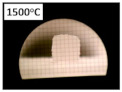	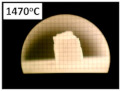	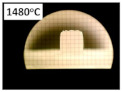

**Table 5:** Phases present in raw material–slag mixtures, after pellet test at 1100 °C and 1400 °C.

Temperature	FMC 45 + Z1	FMC 57 + Z1	PAK + Z1	SP AM 70 + Z1
1100 °C	Solid solution (Mg,Fe)(Fe,Cr,Al)_2_O_4_			Spinel MgAl_2_O_4_
	Periclase MgO		Fayalite Fe_2_SiO_4_	Lead Oxide PbO
	Forsterite Mg_2_SiO_4_		Lead silicate Pb_2_SiO_4_	Forsterite Mg_2_SiO_4_
	Magnesium aluminum oxide		-	Tenorite CuO
1400 °C	Solid solution (Mg,Fe)(Fe,Cr,Al)_2_O_4_			Spinel MgAl_2_O_4_
	Periclase MgO		Fayalite Fe_2_SiO_4_	Forsterite Mg_2_SiO_4_
	Forsterite Mg_2_SiO_4_		-	Güggenite Cu_2_MgO_3_

**Table 6:** EDS analysis in microareas of mixture FMC 45 + slag after corrosion, in the points marked in Figure 7, together with corresponding phases.

*Figure No.*	*Point*	*Phase*	Chemical Composition, wt./mol% *								
			Pb	Cu	Mg	Cr	Al	Si	Fe	As	O
Figure 7a	1	Fe-Cr-O solid solution	1.1/0.2	-	1.2/2.3	12.2/10.8	0.9/1.5	2.7/4.2	70.0/57.9	-	4.3/12.5
	2	-	16.4/2.4	1.0/0.5	21.6/26.6	6.1/3.5	2.9/3.2	3.3/3.5	18.4/9.9	-	23.8/44.5
	3	-	19.0/2.6	2.3/1.0	25.1/28.9	3.8/2.0	1.9/2.0	5.7/5.7	10.1/5.1	-	28.3/49.7
Figure 7b	1	MgO with (Mg,Fe)(Fe,Cr,Al) spinel inclusions	0.8/0.1	6.1/2.3	44.9/44.4	4.6/2.1	1.0/0.9	0.2/0.1	8.4/3.6	1.7/0.6	29.5/44.3
	2	Güggenite s.s.	0.9/0.2	33.1/19.9	18.3/28.8	8.2/6.0	-	1.2/1.6	10.0/6.8	-	10.5/25.0
	3	Mg-rich spinel s.s. Mg-Fe-Cr-Al-O	1.3/0.2	1.3/0.5	15.9/16.7	11.4/5.6	7.4/7.1	1.1/1.0	20.6/9.5	-	34.9/55.9
	4	Cr-rich spinel solid solution Mg-Fe-Cr-Al-O	1.1/0.2	0.9/0.4	16.0/17.5	17.4/8.9	7.2/7.1	0.3/0.3	16.4/7.8	3.2/1.1	32.5/54.1

* The remaining elements are in minor amounts: Na, P, S, Ti, Ca, and Mn. s.s.—solid solution.

**Table 7:** EDS analysis in microareas of mixture FMC 57 + slag after corrosion, in the points marked in Figure 8, together with corresponding phases.

*Figure No.*	*Point*	*Phase*	Chemical Composition, wt./mol% *								
			Pb	Cu	Mg	Cr	Al	Si	Fe	As	O
Figure 8a	1	MgO with (Mg,Fe)(Fe,Cr,Al) spinel inclusions	1.1/0.1	5.5/2.0	51.7/50.2	2.7/1.2	0.8/0.7	0.6/0.5	6.4/2.7	1.8/0.6	27.5/40.6
	2	Fe-rich spinel s.s.Mg-Fe-Cr-Al-O	1.0/0.1	2.7/1.2	16.5/18.9	13.5/7.2	5.5/5.6	0.1/0.1	30.2/15.0	-	29.1/50.6
	3	MgO s.s.	-	3.4/1.1	56.4/49.4	0.9/0.4	-	0.3/0.2	2.5/1.0	-	35.4/47.2
	4	-	33.2/6.0	2.8/1.6	17.4/26.7	6.4/4.6	0.8/1.1	5.9/7.8	4.3/2.9	4.1/2.0	15.8/37.0
	5	Fe-rich spinel s.s.Mg-Fe-Cr-Al-O	6.1/0.8	1.5/0.6	12.2/13.6	9.7/5.1	4.2/4.2	-	23.4/11.3	-	32.4/54.8
Figure 8b	1	MgO with spinel inclusions	0.4/0.1	0.7/0.3	30.7/32.4	15.2/7.5	4.6/4.4	0.6/0.5	16.0/7.4	-	28.6/45.8
	2	Cr-rich spinel s.s.Mg-Fe-Cr-Al-O	0.4/0.1	0.7/0.3	15.6/17.2	32.5/16.7	7.0/6.9	0.6/0.6	5.8/2.8	1.9/0.7	31.6/52.6
	3	Spinel s.s. Mg-Fe-Cr-Al-O	-	1.4/0.6	17.2/19.7	23.6/12.7	7.2/7.4	0.2/0.2	20.0/10.0	-	27.1/47.2

* The remaining elements are in minor amounts: Na, P, S, Ti, Ca, and Mn. s.s.—solid solution.

**Table 8:** EDS analysis in microareas of mixture PAK + slag after corrosion, in the points marked in Figure 9, together with corresponding phases.

*Figure No.*	*Point*	*Phase*	Chemical Composition, wt./mol% *								
			Pb	Cu	Mg	Cr	Al	Si	Fe	As	O
Figure 9a	1	Cr-rich spinel solid solution Mg-Fe-Cr-Al-O	-	5.8/2.7	10.7/13.2	40.1/23.0	8.1/8.9	0.4/0.4	3.6/1.9	3.5/1.4	25.1/46.7
	2	Lead silicate Pb_2_SiO_4_ s.s.	51.8/11.7	3.5/2.6	1.9/3.7	2.1/1.9	3.4/5.9	13.7/22.8	2.7/2.3	-	13.5/39.5
	3	-	19.5/3.3	4.6/2.6	7.1/10.3	20.4/13.8	4.0/5.2	5.3/6.6	13.9/8.8	-	20.7/45.6
Figure 9b	1	Olivine (Ca,Mg,Fe)_2_SiO_4_ **	0.4/0.4	0.5/0.2	9.6/9.8	1.5/0.7	3.9/3.6	25.6/22.6	6.9/3.1	-	30.5/47.1
	2	Lead silicate Pb_2_SiO_4_ s.s.	35.9/6.3	2.7/1.5	1.7/2.5	2.9/2.0	6.0/8.1	19.5/25.3	3.1/2.0	-	19.4/44.2
	3	Cr-rich spinel s.s. Mg-Fe-Cr-Al-O	-	4.9/2.5	9.9/13.0	44.3/27.2	4.0/4.7	-	9.4/5.4	2.1/0.9	22.1/44.1
	4	Fe,Cr-rich spinel s.s. Mg-Fe-Cr-Al-O	1.7/0.4	7.1/4.8	6.1/10.6	32.9/27.0	2.4/3.7	0.9/1.3	34.6/26.4	2.7/1.5	7.8/20.9

* The remaining elements are in minor amounts: Na, P, S, Ti, Ca, and Mn. ** Additionally, 18.6 wt.%/8.3 mol.% of Ca was detected, which substitutes Mg or Fe in olivine. S.s.—solid solution.

**Table 9:** EDS analysis in microareas of mixture PAK + slag after corrosion, in the points marked in Figure 10, together with corresponding phases.

*Figure No.*	*Point*	*Phase*	Chemical Composition, wt./mol% *								
			Pb	Cu	Mg	Cr	Al	Si	Fe	As	O
Figure 10a	1	Spinel MgAl_2_O_4_ s.s.	0.7/0.1	0.6/0.2	18.1/16.4	0.5/0.2	43.8/35.8	0.3/0.2	0.7/0.3	-	33.0/45.5
	2	-	5.3/0.7	15.3/6.2	23.0/24.2	0.3/0.2	2.0/1.9	14.5/13.2	2.0/0.9	-	30.5/48.9
Figure 10b	1	Spinel MgAl_2_O_4_ s.s.	-	1.3/0.5	16.7/15.5	0.5/0.2	40.5/33.7	0.2/0.2	5.1/2.0	-	33.2/46.6
	2	Güggenite s.s.	-	25.0/10.4	41.5/45.3	0.2/0.1	0.4/0.4	-	4.2/2.0	-	23.3/38.6

* The remaining elements are in minor amounts: Na, P, S, Ti, Ca, and Mn. s.s.—solid solution.

## Data Availability

Data available on request due to restrictions eg. privacy or ethical The data presented in this study are available on request from the corresponding author. The data are not publicly available due to technical or time limitations.
